# Explicit underdose control based on toxicity: Four points to consider

**DOI:** 10.1002/sim.9492

**Published:** 2022-11-17

**Authors:** Paul H. Frankel, Elizabeth Garrett‐Mayer, Mark D. Krailo

**Affiliations:** ^1^ Department of Computational and Quantitative Medicine City of Hope Duarte California USA; ^2^ Center for Research and Analytics, American Society of Clinical Oncology Alexandria Virginia USA; ^3^ Department of Population and Public Health Sciences Keck School of Medicine, University of Southern California Los Angeles California USA

There are many design options for toxicity‐driven phase 1 dose‐finding in oncology drug development. There are methods that are simple, traditional and transparent, such as the 3 + 3^1^ or rolling 6 design^2^ that do not target a dose limiting toxicity (DLT)‐rate and are focused on exploring escalating doses subject to limits on patient risk. On the other end of the spectrum are designs that focus on a DLT target‐rate and require sophisticated tools throughout the conduct of the study, are less transparent, and require statistical support. Two examples are the CRM design^3^, ^4^ and the BLRM design.^5^ There are also designs between these two extremes. Closest to the 3 + 3 and rolling 6 are queue‐based variations of those designs^6^ or other A + B designs^7^ that also explore doses subject to pre‐specified rules to limit patient risk. Closer to the CRM or BLRM are statistically motivated methods such as the mTPI,^8^ TEQR,^9^ and BOIN,^10^ where there is a target DLT rate and/or range, but simple decision rules and tables reduce the absolute requirement for sophisticated tools or a statistician except in the design and final analysis.

Entering into this challenging area is the current effort^11^ to provide a new twist to the BLRM. The key change is to augment the BLRM with an underdose control rule to be pitted against overdose control. The popularity of this method will likely be limited, as this method is being introduced exactly at the time when the “more is better” paradigm is less accepted.^12^ Project Optimus, led by the FDA, is specifically concerned with the assumption that the maximum tolerated dose provides the best toxicity‐efficacy trade‐off, leading to a focus on selecting doses with relatively high toxicity. Instead, low toxicity is not generally an issue to be used to eliminate certain doses for possible consideration for future study. Nevertheless, there are increasingly rare, but important, clinical settings where underdose control concerns persists and this is exactly where such a tool can be considered. Primarily, these are short‐term therapies with curative intent.

Once restricted to such a setting, is this BLRM with both overdose control and underdose control superior to other designs that seek to find a DLT rate within a pre‐specified range? The authors make the reasonable case (see Table 7 of Reference 11) that in some settings, depending on the details of the dose‐toxicity curve and the dose increment, this method may have some advantages over some alternative DLT‐target rate designs. In other settings, it may not. Knowing when and how to use a new tool is critical for statisticians in the field, and to that end, we will consider 4 separate points.

The first point is the concern expressed in Table 1 (see Reference 11), where 0/3 DLTs are observed at each dose level of 10 mg, 25 mg, 50 mg and 100 mg, and yet 200 mg exceeds the overdose control rule so the next patients are not treated at that dose. At first view, this might appear to convey a problem since no escalation is permitted with 0 DLTs on multiple dose levels. However, it is very uncommon in a traditional 3 + 3 design to see dose doubling between a fourth and fifth dose level. The BLRM with overdose control may be pointing to this concern in the design when encountering the 100 mg increment in this case. The authors did not present the full BLRM model behind this hypothetical data scenario, but would a lesser increment, such as a 40 mg increment, representing a 40% increase, have been allowed? This is not to detract from the main role of underdose control, but simply to highlight that the concern about **the conservative nature of the BLRM design with overdose control may have been overstated and this may influence the choice of method**.

The second point to highlight relates to the details on how to use this modified BLRM method. The BLRM method is usually written as a guide while providing certain constraints on decision‐making. It presents a permissible range, and a guide within that range, and otherwise, the physicians use all the information at their disposal to make a dosing decision for the next patient or cohort. BLRM with underdose control should be implemented in a manner that benefits from this history and inherits that culture. The BLRM designs are generally implemented as proscriptive, not prescriptive, which is a considerable advantage. **This type of flexibility can be written into other design choices, but the BLRM designs lend themselves to permitting physician judgment within certain limits, and modifications of the BLRM should inherit this culture.**

The third point relates to the interaction with physicians. Physician judgment, and especially the principal investigator (PI), is key to the choice of the design and design details. For the BLRM or related methods, this includes the DLT definition, the DLT‐target, and the DLT‐target acceptable range which are all very related. There is also the selection of priors and dose increments and other choices required from the clinical team that drive such models.

Usually, a DLT is declared when a patient encounters toxicities that are considered at least a) severe (eg, grade 3 per CTCAE) and beyond readily reversible events that can be managed by pre‐planned dose modifications or b) life‐threatening (eg, grade 4) adverse events, or c) adverse events such that the patient cannot tolerate continued treatment (eg, delays>2 weeks). The primary patient‐level quantity observed in a phase 1 trial is a simple binary DLT (Y/N) call usually based on the first cycle of therapy. Consideration of near DLTs or particularly devastating DLTs, inconsistent DLTs across patients with debatable attribution to treatment, later cycle toxicity and biological correlatives (eg, PK/PD) all require PI judgment (or more generally the dose level review committee/study team). Statistical rules provide a guide and are also required by IRBs to restrict those decisions so no PI can use their judgment to put patients at unbounded risk.

This raises an interesting question: When is it necessary to prevent underdosing by a statistical rule? If the PI, who may have been concerned with underdosing a priori prefers not to escalate based on their judgment, is anything gained by a statistical underdosing rule based on a limited Bayesian framework created by abstract queries of the PI? The PI can review the actual data at hand and use their judgment which is always layered upon their prior experience and knowledge. If there are no DLTs, but every patient experienced moderate toxicity, that may be too high a dose in some settings. Beyond this consideration, the designation of underdosing presumes that the hypothesized clinical efficacy increases with increased dose levels; this generally cannot be confirmed in a phase 1 setting. Ultimately, if the target DLT‐rate is 25%, the most commonly selected target, do the physicians need to have an acceptable interval of 0.16 to 0.33 or would it be just as reasonable to set the range as 0% to 33% DLT rate for the permissible doses? By identifying a set of doses that are tolerable, the doses to be tested for future investigation can be chosen by supplementary information, such as a measure of the putative biological mechanism of action and number of cycles tolerated. The candidate dose (or doses) for further study would then not be restricted to a mathematically framed underdosing rule. As a result, this trade‐off between underdosing and overdosing as proposed may find more applications where it is reframed to be a concern for an excessive risk of two competing adverse events such as DLT vs graft‐rejection that are expected to be present on different ends of the dose ladder on a graft‐rejection drug and where simulation studies are often used to help modify rule‐based designs.^13^ **The clinical settings where a minimum DLT percentage must be pre‐specified are very limited, and this should be carefully discussed.**

The fourth point to address is the statement^11^ that “Simulation results reveal that the new designs have better accuracy and treat more patients at the MTD.” This is a common statement seen in DLT‐target methods, but why is it good to treat more patients at the MTD? The MTD is not necessarily the recommended dose, especially if targeting a 25% DLT rate. This can be compared to the traditional 3 + 3 rules where empirically 0 or 1 in 6 experience a DLT (0%‐16.7%) at the MTD. And if the target was thought to be the recommended phase 2 dose (RP2D), how would the PI have any expectation that any pre‐specified target DLT rate identifies a good RP2D? Does accuracy on an arbitrary target matter? There are no clinical data to support any target DLT‐rate.^14^ Today, the BLRM original first author prefers to specify a “range of acceptable toxicity, for example a 0‐15%” DLT rate, depending on the clinical setting (Beat Neuenschwander, personal communication), and tutorials from more than a decade ago when there was less targeted therapy than today already suggested 10%‐20% might be more appropriate for treatments given over many cycles.^4^ This is also consistent with a physician survey raising concerns about DLT‐rate selection and related simulations.^15^ **Users of these methods need to make sure not to conflate the MTD with candidate recommended phase 2 doses.**

That any such designs are appropriate in only some very select settings does not detract from the effort. The method is both thought provoking and may provide some advantages over competing designs in certain situations. Inheriting the culture and flexibility of the BLRM method is one of its key strengths. Clinical judgment and flexibility can improve the actual reproducibility of phase I studies and improve the selection of the candidate dose (or doses) to further explore, even if mathematical reproducibility suffers. This is because the same patients, coming in the same order is never possible. It is challenging to convey such fuzzy logic, so at this point it will be left as just an opinion.

## Data Availability

Data sharing is not applicable to this article as no new data were created or analyzed in this study.
